# Comparison of Pregabalin and Midazolam as Premedication in Children Undergoing General Anesthesia for Dental Treatment

**DOI:** 10.5812/aapm-149486

**Published:** 2024-12-15

**Authors:** Maryam Hajiahmadi, Nasser Kaviani, Elahe Asnaashari Esfahani, Sanaz Rajaee

**Affiliations:** 1 Department of Pediatrics, Dental Research Center, Dental Research Institute, School of Dentistry, Isfahan University of Medical Sciences, Isfahan, Iran; 2 Department of Oral and Maxillofacial Surgery, Dental Research Center, Dental Research Institute, School of Dentistry, Isfahan University of Medical Sciences, Isfahan, Iran; 3 Department of Pediatrics, School of Dentistry, Isfahan University of Medical Sciences, Isfahan, Iran

**Keywords:** Pregabalin, Midazolam, Premedication, General Anesthesia, Dental Treatment

## Abstract

**Background:**

Pediatric dentists employ both pharmacological and non-pharmacological behavior control methods. Despite the use of behavioral control techniques, some young children cannot undergo treatment in the office, making sedation or general anesthesia necessary. Premedication drugs can be used before general anesthesia to reduce anxiety, control pain, induce amnesia, prevent nausea, and avert potential complications. The search for the ideal premedication for children is ongoing.

**Objectives:**

This study aims to compare the effects of pregabalin and midazolam (MID) in children undergoing dental treatment under general anesthesia.

**Methods:**

This prospective, triple-blind study included 64 children aged 2 - 6 years who required dental treatment under general anesthesia. Participants who met the inclusion criteria were enrolled. One group of children received pregabalin syrup, while the other group received MID syrup. The comfort of the child during separation from the parents, ease of venous access, and degree of sedation upon entering the operating room were evaluated. Blood pressure, heart rate, and blood oxygen levels were measured at baseline and every 30 minutes thereafter. Additionally, the duration of the patient's stay in recovery until discharge was recorded and compared between the two groups. Statistical analyses were performed using chi-square, Mann-Whitney U, Fisher's exact test, and SPSS version 14 software.

**Results:**

No statistically significant differences were found between premedication with MID and pregabalin in terms of anxiety during venous access, parental separation anxiety, restlessness in recovery, duration of recovery stay, or changes in heart rate, blood pressure, and blood oxygen levels between the two groups. However, a statistically significant difference was observed between the two groups regarding the degree of sedation before entering the operating room.

**Conclusions:**

Both pregabalin and MID were effective for premedication in terms of sedation and anxiety reduction, with no significant difference between the two drugs in these outcomes.

## 1. Background

One of the primary challenges in pediatric dentistry is managing anxious and uncooperative children (1). One of the main causes of children's non-cooperation is the fear of dental procedures (2). Pediatric dentists must use both pharmacological and non-pharmacological behavior control methods to provide effective and safe treatments (3). Non-pharmacological methods include techniques such as tell-show-do, voice control, non-verbal communication, positive reinforcement, presence of parents, and distraction (3). However, despite the use of these behavioral control techniques, some young children, as well as those with physical, psychological, mental, and emotional issues, cannot be treated in the office. Therefore, pharmacological options are considered for sedation or general anesthesia (4).

Premedication agents are used prior to general anesthesia (5). These drugs serve to reduce anxiety, control pain, induce amnesia, prevent nausea, reduce secretions, and provide prophylaxis for potential complications. Premedication is typically administered shortly before anesthesia (5). The most commonly used prophylactic agent in children is midazolam (MID) (6). The rapid onset and relatively short duration of action of MID make it a useful agent for reducing preoperative anxiety and facilitating separation from parents, with minimal unwanted side effects (6, 7). Midazolam, a sedative and hypnotic anti-anxiety drug, is widely used as a prophylactic agent in various settings (7). However, high doses of MID can cause hypoxia and hypoventilation. In some cases, respiratory depression has been observed in adults, though reports in children are limited. Reported side effects of MID include restlessness, hyperactivity, and involuntary movements (7).

Pregabalin is another drug that can be used as a prophylactic agent. It has anti-anxiety, analgesic, and anticonvulsant properties (8) and is commonly used in the treatment of neuropathy, fibromyalgia, pain associated with diabetic peripheral neuropathy, postoperative neuroallergy, and partial epilepsy (1, 9, 10). The most common side effects are confusion and drowsiness (9). Due to its analgesic, anti-anxiety, and sedative effects, pregabalin is also used in premedication for children's anesthesia (9).

Although pregabalin is a structural analog of GABA, it does not directly bind to GABA-A, GABA-B, or benzodiazepine receptors. Instead, pregabalin binds to the alpha-2-delta subunit of calcium channels (10, 11) and increases the activity of glutamic acid decarboxylase. By binding to the subunits of calcium channels, it reduces the calcium influx caused by depolarization, thereby decreasing or inhibiting the release of excitatory neurotransmitters such as glutamate, noradrenaline, and substance P (12, 13). 

When taken orally, pregabalin has a bioavailability of ≥ 90%, reaching its peak concentration 0.7 - 1.5 hours after administration. More than 98% of the drug is excreted unchanged in the urine (12, 14-16). If taken with food, the absorption rate remains unchanged, but the peak plasma concentration is reduced, and the time to reach peak concentration may be delayed by up to 3 hours (12, 14, 16). 

In a 2012 study by Ghai et al. in India on the use of pregabalin and gabapentin as premedication before surgery, both drugs significantly reduced preoperative anxiety and improved sedation before the procedure, without causing side effects (17). 

In a 2015 study by Eskandarian et al. in Shiraz on the effectiveness of pregabalin in the dental treatment of anxious children, it was shown that pregabalin was a safe and effective drug that improved children’s behavior control and the success of dental treatment. The sedative and anti-anxiety effects of this drug were observed two hours after oral administration, with no significant side effects (1). 

In a 2018 study by Marouf in Egypt, pregabalin premedication for children undergoing adenotonsillectomy with sevoflurane anesthesia was found to reduce postoperative vomiting. Additionally, the use of pregabalin did not affect the time to eye opening or the duration of stay in the post anesthesia care unit (PACU) (18).

In a 2021 study by Talaat and El-Gendy in Egypt, premedication with pregabalin, compared to MID, resulted in children opening their eyes more quickly during recovery, and the length of stay in the PACU was reduced (19). 

Midazolam is the standard drug used as premedication for children, but it has side effects, such as breathing problems, which have raised concerns among doctors regarding its use. Additionally, the drug has a very bitter taste, which reduces children's cooperation when taking it. Since pregabalin has a more pleasant taste and has been used in similar studies with children, with the most common side effects being confusion and drowsiness, it appears that pregabalin could provide sedation similar to MID, with fewer side effects.

## 2. Objectives

As the effects of pregabalin in pediatric dentistry have not been thoroughly studied, the present study aimed to investigate the effects of pregabalin premedication on the complications of general anesthesia in pediatric dentistry procedures.

## 3. Methods

After obtaining ethics approval from the Ethics Committee of Isfahan University of Medical Sciences, children aged 2 - 6 years who were referred to a private dental clinic in Isfahan, Iran, and were classified as ASA I or ASA II, were included in the study. Children who were in Frankl categories 1 (refusal of treatment, crying forcefully, fearful, or showing other overt signs of extreme negativism) or 2 (reluctant to accept treatment, uncooperative, showing some evidence of negative attitude but not pronounced, e.g., sullen, withdrawn) in terms of cooperation, and were candidates for dental treatment under anesthesia, were also included in the study.

### 3.1. Exclusion Criteria

Patients were excluded from the study if they lacked the ability to cooperate, or if their parents did not consent to the study. Additionally, children with uncontrolled systemic diseases, a history of epilepsy, neuropathic pain, convulsive disorders, mental or physical disabilities, digestive problems, hepatic insufficiency, or those taking medications such as benzodiazepines, anticoagulants, barbiturates, or painkillers were excluded, as these medications could interfere with the effects of the study drug. Children with contraindications for general anesthesia or those requiring tooth extraction were also excluded from the study.

In this triple-blinded, prospective clinical trial, all individuals involved in the study (the dentist, the patients' parents, the data recorder, and the statistical consultant) were blinded to which medication each child received. An anesthesiologist not involved in the study instructed the parents to keep their child fasting for 6 hours before premedication (1). The study included 64 children aged 2 - 6 years (calculated using a statistical formula). Before the study, the procedural steps, drugs used, and their potential benefits and risks were explained to the parents or guardians, and informed consent was obtained.

A convenient sampling method was used to divide the children into two groups: Experimental and control. In this method, each child was randomly assigned a number, and a person not involved in treatment or data recording selected the children using a random number table, resulting in 32 children in the pregabalin group and 32 children in the MID group.

For the comparative study of the premedication effects of pregabalin and MID on children undergoing dental treatment under anesthesia, with a significance level of 5% (α = 0.05) and a test power of 80% (β = 0.2), based on the study by Talaat and El-Gendy (19), the sample size was calculated to detect a difference of up to 70% standard deviation (δ = 0.7 σ) using the following formula:


$$n\geq \frac{2\sigma^{2}{\left(Z_{1-\frac{\alpha }{2}}+Z_{1-\beta }\right)}^{2}}{\delta^{2}}=\frac{2{\left(1.96+0.84\right)}^{2}}{{\left(0.7\right)}^{2}}=32$$

**Figure 1.**
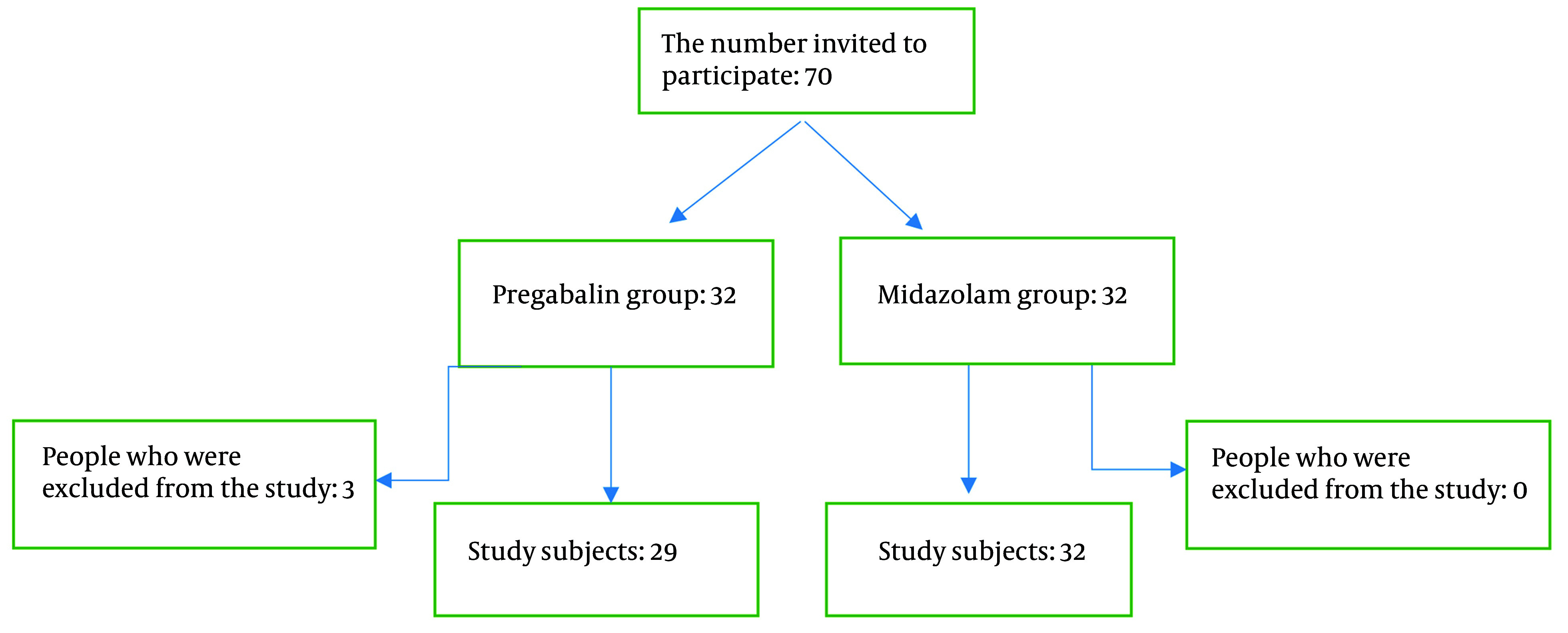
Flow diagram of participants.

There were 32 participants in each group. In the MID group, all participants remained in the study, while in the pregabalin group, 3 participants were excluded due to the need for tooth extraction (Figure 1). Dental treatments in both groups were provided by pediatric dental specialists. 

In the test group, 100-mg/5 mL pregabalin syrup (Sobhan Co., Iran) was administered at a dose of 5 mg/kg (18) for each child. In the control group, MID syrup (Eksir Co., Iran) was used at a dose of 0.75 mg/kg in a total volume of 15 mL (19). Both drugs were administered half an hour before venipuncture (18-20). 

We used the following four criteria to assess the convenience of venipuncture and anesthesia induction in children: Cries, shouts, limb movement, and no reaction (20). After the child entered the operating room and was separated from the parents, the Parental Separation Anxiety Scale (PSAS) was used to assess the child's anxiety during separation (21) (Table 1). 

**Table 1.** Parental Separation Anxiety Scale

Score	Definition
**1**	The child is easily separated from the parents
**2**	The child moans but is easily separated from the parents and is not attached to them
**3**	The child cries, is consoled with difficulty, and is attached to the parents

Additionally, the degree of the child's sedation upon entering the operating room was measured. The criteria for this measurement are as follows (22) (Table 2). 

**Table 2.** Sedation Scale

Score	Definition
**1**	The child is anxious and listless
**2**	The child is calm, cooperative, and concentrated
**3**	The child is lethargic and responsive to verbal commands
**4**	The child is not responsive to verbal commands but responsive to painful stimuli

In both groups, general anesthesia was administered intravenously using sodium thiopental (Kavosh Gostar Co., Iran) at a dose of 1 mg/kg, fentanyl (Caspin Co., Iran) at a dose of 1 µg/kg, and atracurium (Aboureihan Co., Iran) at a dose of 0.8 mg/kg, followed by tracheal intubation. After induction, anesthesia was maintained using a combination of oxygen and nitrous oxide (at a 50:50 ratio) and 1% isoflurane (Primal Co., India). The child was connected to the anesthesia machine, and the necessary dental treatments were performed. To ensure homogeneity, none of the patients were administered local anesthetic agents. 

Before the procedure, after intubation, and during the procedure, the child’s heart rate and blood pressure were monitored every 30 minutes. The type and duration of the dental procedure were recorded for each child.

After the procedure, the child was transferred to the PACU bed, and the parents joined the child. The child’s post-anesthetic medical complications, including nausea and vomiting (whether they occurred or not until discharge), were assessed. The time of eye opening was recorded for each child. The need for analgesics after the procedure until discharge was noted (whether required or not). The time from the patient’s arrival in the recovery bed to discharge was also recorded. Additionally, side effects such as nausea, vomiting, and hypotension during recovery were evaluated. The variables evaluated in both groups were analyzed separately and then compared.

### 3.2. Data Analysis 

The analysis was conducted at both descriptive and inferential levels. At the descriptive level, frequency and percentage indicators were reported for qualitative variables, and means and standard deviations were reported for quantitative variables. Statistical graphs were also created for the variables. At the inferential level, the normality of the data was assessed using the Shapiro-Wilk test. If the data followed a normal distribution, ANOVA and independent *t*-tests were applied. If the data did not follow a normal distribution, the Mann-Whitney and Kruskal-Wallis tests were used. Additionally, chi-squared and binomial tests were employed to analyze qualitative variables. All tests were performed at a 5% significance level using SPSS version 26.

## 4. Results

The two groups were compared in terms of gender and duration of anesthesia. According to Table 3, the two groups showed no significant difference in gender distribution and duration of anesthesia.

**Table 3.** Gender Distribution and Duration of Anesthesia in the Two Groups

Vriables	Midazolam	Pregabalin
**Gender (male/female) (%)**	50/50	48.3/51.7
**Mean duration of general anesthesia**	55.78 ± 17.65	58.79 ± 30.19

Nine people in the MID group and 14 people in the pregabalin group cried. One person in each group screamed, while 7 people in the MID group and 6 people in the pregabalin group moved their limbs. Finally, 15 people from the MID group and 8 people from the pregabalin group did not react. There was no statistically significant difference between the two groups in terms of the level of anxiety during venipuncture (P-value = 0.368) (P > 0.05).

There was no statistically significant difference in the children’s comfort level when separated from their parents between the two groups, as shown in Table 4 (P > 0.05).

**Table 4.** Children’s Comfort Level

Separation From the Parents	Intervention ^a^		X^2^	P-Value
	Midazolam	Pregabalin		
**Score 1**	22 (68.8)	16 (55.25)	2.606	0.272
**Score 2**	9 (28.1)	9 (31.0)		
**Score 3**	1 (3.1)	4 (13.8)		
**Total**	32 (100)	29 (100)		

^a^ Values are expressed as No. (%) unless otherwise indicated.

The amount of sedation before entering the operating room was statistically significantly different between the two groups (P < 0.05). The average scores in the two groups revealed that the degree of sedation in the MID group was higher than in the pregabalin group (Table 5). 

**Table 5.** Sedation Before Entering the Operating Room

Sedation	Intervention ^a^		X^2^	P-Value
	Midazolam	Pregabalin		
**Score 1**	4 (12.55)	12 (41.4)	15.289	0.000
**Score 2**	14 (43.8)	16 (55.25)		
**Score 3**	14 (43.8)	1 (3.4)		
**Total**	32 (100)	29 (100)		

^a^ Values are expressed as No. (%) unless otherwise indicated.

The two groups did not show a statistically significant difference in terms of restlessness during recovery (P > 0.05), and neither of the drugs caused significant changes in heart rate from the moment of induction to the end of recovery. Additionally, the two drugs did not differ significantly in terms of changes in heart rate (P > 0.05).

Based on Table 6, MID caused significant fluctuations in systolic blood pressure (P-value = 0.039). However, in the pregabalin group, the drug did not cause any significant changes (P-value = 0.671). Nevertheless, the two drugs were not significantly different in inducing changes in systolic blood pressure (P-value = 0.776) (P > 0.05).

**Table 6.** Hemodynamic Factors and Their P-Values

Hemodynamic Factor	P-Value
**Heart rate**	0.432
**Systolic blood pressure**	0.776
**Diastolic blood pressure**	0.203
**Blood oxygen saturation**	0.427

In both the MID and pregabalin groups, there were significant changes in diastolic blood pressure fluctuations. However, the two drugs did not differ significantly in terms of diastolic blood pressure changes (P-value = 0.203) (P > 0.05).

Both drugs caused significant changes in blood oxygen saturation. However, a comparison of the two drugs showed no statistically significant difference in blood oxygen changes (P-value = 0.427) (P > 0.05).

The average duration of stay in the recovery room was not significantly different between the two groups (P-value = 0.650). That is, pregabalin premedication, compared to MID, did not decrease or increase the recovery time (P > 0.05).

Additionally, side effects such as nausea, vomiting, bradycardia, and blood pressure drops during recovery were evaluated in both groups. None of these side effects were observed in the patients during recovery.

## 5. Discussion

This study aimed to compare the effectiveness of premedication with pregabalin and oral MID in children who were candidates for dental treatment under general anesthesia. As mentioned in the previous section, there were no statistically significant differences between premedication with MID and pregabalin in terms of anxiety during venipuncture, ease of separation from parents, restlessness during recovery, duration of stay in the recovery room, or changes in heart rate, blood pressure, and blood oxygen. However, the two groups did show a statistically significant difference in the degree of sedation before entering the operating room. Additionally, both drugs caused significant changes in blood pressure and oxygen levels during general anesthesia. In this section, we compare the results of this study with previous studies.

A study by Hill et al., conducted in Germany in 2000, investigated the use of pregabalin in patients experiencing pain after dental treatment. The study found that pregabalin significantly reduced pain intensity and increased the duration of pain relief compared to a placebo. Furthermore, when compared to ibuprofen, pregabalin provided a longer duration of pain relief. In the present study, pregabalin reduced restlessness during recovery, with no significant difference compared to the standard drug, MID (23).

In a study by Paech et al. in 2007 in Australia on the use of pregabalin premedication to reduce pain after minor gynecological surgeries, the incidence of light-headedness, visual impairment, and difficulty walking after discharge was significantly higher in the pregabalin group. However, in our study, no side effects were observed in any of the patients. Additionally, in Paech et al.'s study, no difference in postoperative pain relief was found between the pregabalin group and the placebo group, which contrasts with our study, where pregabalin reduced patients' restlessness. One reason for these inconsistent results could be the differences in the treatments rendered in the two studies. In Paech et al.'s study, gynecological surgery was performed, whereas dental treatments were rendered in our study. Furthermore, pregabalin was administered based on the child's weight in the present study, while in Paech et al.'s study, all patients received 100 mg of pregabalin. In our study, 64 samples were included, but Paech et al.'s study involved 90 patients, which might explain the differences in results (24).

A study by White et al. in 2008 in the United States found that administering pregabalin before surgery improved sedation, which is consistent with the present study. However, in White et al.’s study, pregabalin did not affect preoperative anxiety, postoperative pain, or the recovery process after surgery. These findings contrast with our study, where pregabalin reduced preoperative anxiety and restlessness during recovery. The differences in results may be due to the distinct age groups in the two studies. In the present study, the participants were between 2 and 6 years old, while Paul’s study included subjects aged 18 to 70. Additionally, in White et al.’s study, the subjects randomly received either a placebo or 75 - 300 mg of pregabalin, whereas in our study, the dosage was based on the child’s weight. Moreover, objective criteria were used to assess preoperative anxiety in our study, while in White et al.’s study, the criteria were subjective and relied on the patients' opinions (8).

In a study by Gonano et al. in 2011 in Australia, which investigated the use of pregabalin in patients undergoing minor orthopedic surgeries, pregabalin reduced preoperative anxiety and postoperative pain without causing side effects such as dizziness or increasing the stay in the post-anesthesia care unit. These findings are consistent with the present study (25).

A 2012 Indian study by Ghai et al. showed that administering pregabalin and gabapentin as premedications before surgery significantly reduced preoperative anxiety and improved preoperative sedation without side effects, which aligns with the present study's results (17).

According to a study by Iftikharian et al. in 2013 in Shiraz, administering pregabalin before wisdom tooth surgery significantly reduced systolic and diastolic blood pressure and effectively reduced postoperative pain scores. In the present study, pregabalin caused changes in diastolic blood pressure but did not result in significant changes in systolic blood pressure or heart rate. Additionally, while Iftikharian et al.’s study found that pregabalin reduced postoperative pain, the present study observed a decrease in patients' restlessness during recovery (26).

In a study by Faghihian et al. in 2017 in Isfahan, which compared the effects of MID and melatonin premedication in children under anesthesia for dental treatment, MID was found to be superior to melatonin in terms of pre-anesthesia sedation and ease of venipuncture, as well as decreasing the need for analgesics after the procedure. However, in the present study, MID and pregabalin were not significantly different in terms of anxiety during venipuncture, comfort during separation from parents, or restlessness during recovery. The two drugs only differed in the degree of sedation before entering the operating room, with MID being superior to pregabalin in this regard (20).

In a study by Marouf in 2018 in Egypt, pregabalin premedication for children undergoing adenotonsillectomy with sevoflurane anesthesia reduced postoperative vomiting. Additionally, administering pregabalin premedication did not affect the eye-opening time or the length of stay in the PACU. In the present study, pregabalin and MID premedication did not significantly differ in terms of recovery room duration, and no nausea or vomiting was observed in any of the patients during recovery (18). 

In a 2019 study by Nimmaanrat et al. in Thailand, which examined the anti-anxiety effects of pregabalin and diazepam premedication compared to placebo, it was found that neither diazepam nor pregabalin was superior to the other. However, both failed to demonstrate an anxiolytic effect compared to placebo, despite increasing the level of sedation. This suggests that pregabalin and diazepam are not the anxiolytic drugs of choice for premedication in patients scheduled for elective surgery. These results are contrary to our study, as both pregabalin and MID served as anti-anxiety drugs, and, similar to the above study, they increased the degree of sedation before entering the operating room. The differences in results between the two studies may be due to the age groups involved; in the Nimmaanrat et al.’s study, the participants were aged 18 - 70 years, while in the present study, the drugs were administered based on the child’s weight, 30 minutes before the procedure. In contrast, Nimmaanrat et al.’s study administered drugs with a fixed dose once the night before the surgery and again 2 hours before venipuncture (27).

A systematic study by Torres-Gonzalez et al. in 2020 in Spain showed that using pregabalin before treatment to control pain can reduce the anxiety of surgical patients and control hemodynamic changes without severe side effects, which is consistent with the present study (28).

In a study by Talaat and El-Gendy in 2021 in Egypt, premedication with pregabalin, compared to MID, resulted in children opening their eyes in a shorter time during recovery and reduced the length of stay in the PACU (29). This differs from the results of the present study, in which the subjects’ duration of stay in the recovery room was not significantly different (19).

In the present study, pregabalin reduced the patients’ restlessness during recovery, which is consistent with the findings of studies by Verma et al. in 2022 (29) in India and Tsai et al. in 2023 (30), in which pregabalin reduced postoperative pain.

This research focused on children in the 2 to 6-year-old age group, the most common group requiring treatment under general anesthesia. The results may be generalized to other pediatric groups. A limitation of this study is the small sample size. Since there were few participants in each group, it is recommended to conduct studies with a larger number of participants.

### 5.1. Conclusions

This study demonstrates that premedication with pregabalin and MID has no statistically significant difference in terms of physiological and sedation factors before entering the operating room. Pregabalin can be considered a suitable alternative to MID as a premedication.

## Data Availability

The dataset used in the study is available upon request from the corresponding author during submission or after publication.

## Clinical Trial Registration Code:

<a href="https://irct.behdasht.gov.ir/trial/67392">IRCT20210419051016N4</a>
